# Case Report: Extensive digital gangrene as a primary manifestation of late-onset systemic lupus erythematosus

**DOI:** 10.12688/f1000research.124225.2

**Published:** 2022-09-07

**Authors:** Soumaya Boussaid, Marouene Ben Majdouba, Sonia Rekik, Samia Jemmali, Khaoula Zouaoui, Safa Rahmouni, Hela Sahli, Mohamed Elleuch

**Affiliations:** 1 Rheumatology Department, La Rabta Hospital, La Rabta Jebbari, Tunis, 1007, Tunisia; 2 Faculty of Medicine of Tunis, University Tunis el Manar, La Rabta Jebbari, Tunis, 1007, Tunisia; 3 Research unit LR 05 SP 01, La Rabta Hospital, La Rabta Jebbari, Tunis, 1007, Tunisia

**Keywords:** Systemic lupus erythematosus, digital gangrene, vasculitis

## Abstract

**Background:** Digital gangrene is a rare but serious complication of systemic lupus erythematosus (SLE). It occurs usually in middle-aged patients with longer disease duration.

**Case:** Herein we report the case of a 56-year-old man (with no history suggestive of Raynaud’s phenomenon, diabetes mellitus, smoking, trauma, infection, or chemical exposure), who presented with SLE and digital gangrene was among the first signs. He presented with a one-month history of joint pain, hair loss, photosensitivity, mouth ulcers, malar rash, dyspnea, and digital pain. Physical examination revealed painful and diffuse erythematous skin lesions in the extremities and back, as well as cyanosis in the fingers. We noted lymphocytopenia (600 cells/mm ^3^), and an elevated C-reactive protein (15.1 mg/l) on laboratory tests. Immunological tests were positive for antinuclear antibodies (ANA) with Title 1:400. Pulmonary computed tomography revealed pulmonary fibrosis, and pulmonary function tests revealed the restrictive pulmonary disease. Diagnosis of SLE with lung involvement was retained. The immunological assessment in search of elements in favor of a vascular origin of the patient’s skin lesions was negative. Treatment was initiated with 200 mg/day hydroxychloroquine. For dermal and pulmonary involvement, intravenous (IV) pulse therapy was used with methylprednisolone (1,000 mg/d for three consecutive days monthly) and cyclophosphamide (1 g/month). Calcium blocking agents were also prescribed. However, the lesions did not improve. The patient was given two infusions of rituximab (1 g) at a 14-day interval with a marked improvement ofthe majority of vasculitis lesions, and a partial improvement of dyspnea.

**Conclusions:** Digital gangrene is a rare complication of late-onset SLE, especially as a primary manifestation.

## Introduction

Systemic lupus erythematosus (SLE) is an autoimmune disease affecting multiple organs and systems, characterized by an autoimmune response to nuclear antigens. Young women are most likely affected by this disease. This condition appears to be rare after the age of 50 years. ^1^ With a cumulative incidence of 1.3%, digital gangrene is a rare but a severe complication of SLE. It is the initial manifestation of the disease in only 0.2% of cases. ^2^^,^^3^ It was initially identified by Dubois ^4^ and Alarcon-Segovia. ^5^ Mechanisms, such as vasculitis, thromboembolism, premature atherosclerosis, vasospasm, and coagulability can contribute to the development of gangrene. ^2^ The major risk is digital amputation. ^2^ Herein, we present a case involving a male patient with extensive digital gangrene as a primary manifestation of late-onset SLE associated with severe and necrotizing vasculitis in the absence of antiphospholipid antibody syndrome (PLAS), which was managed by rituximab.

## Case report

This case involves a 56-year-old retired Caucasian male patient with a one-month history of joint pain, hair loss, photosensitivity, mouth ulcers, malar rash, dyspnea (New York Heart Association (NYHA) stage 3), and digital pain. Physical examination revealed good overall condition and normal vital signs. All peripheral pulses were palpable. Painful and diffuse erythematous skin lesions in the extremities and back, as well as cyanosis in the fingers were noted. Pulmonary auscultation showed small crackles. The rest of the physical examinations were normal. Based on these clinical manifestations, diagnosis of SLE was suspected. Laboratory examinations were therefore carried out and they revealed normal hemoglobin level (Hb=12.1 g/dl) (NV: >12 g/dl) and lymphocytopenia (600 cells/mm ^3^) (NV: 3,000–9,500 cells/mm ^3^). Platelets were normal at 209,000/mm ^3^ (NV: >120,000/mm ^3^). Partial Thromboplastin Time was at 83% (NV: >70%).

The C-Reactive Protein was elevated (15.1 mg/l) (NV: <5 mg/l). Immunological tests were positive for Anti Nuclear Antibodies (ANA) with Title 1:400 (>1/160) speckled and anti-ds DNA dosed by ELISA (with a title of 102,37 UI/ml (NV: 50 UI/ml), anti-SSA 52, and anti-mitochondrial antibody M2. Anti-Smith, anti-SSB, anti-RNP antibodies, and Anti-Neutrophil Cytoplasmic Antibodies (ANCA) were negative. The test of serum Complements (CH50, C3, C4) was normal. Regarding digital pain, simple x-rays were performed and they did not show osteoarticular damage. Concerning dyspnea, pulmonary computed tomography (CT) revealed pulmonary fibrosis, and pulmonary function tests revealed restrictive pulmonary disease. Diagnosis of SLE with lung involvement was retained according to the American College of Rheumatology (ACR) classification criteria for SLE. ^6^ Faced with cutaneous lesions suggestive of vasculitis, we re-examined the patient in search of thromboembolic risk factors. No history suggestive of Raynaud’s phenomenon, diabetes mellitus, smoking, trauma, infection, or chemical exposure was found. The patient had neither a personal nor a family history of thrombotic events. The immunological assessment in search of elements in favor of a vascular origin of the patient’s skin lesions was negative: Prothrombin, anti-Cardiolipin antibody (aCL), Lupus Anticoagulant (AL), antiß2 glycoprotein (GPI), and cryoglobulin levels were negative. Serological tests for hepatitis B and C and human immunodeficiency virus were also negative. The lipid profile was normal.

**Figure 1.**
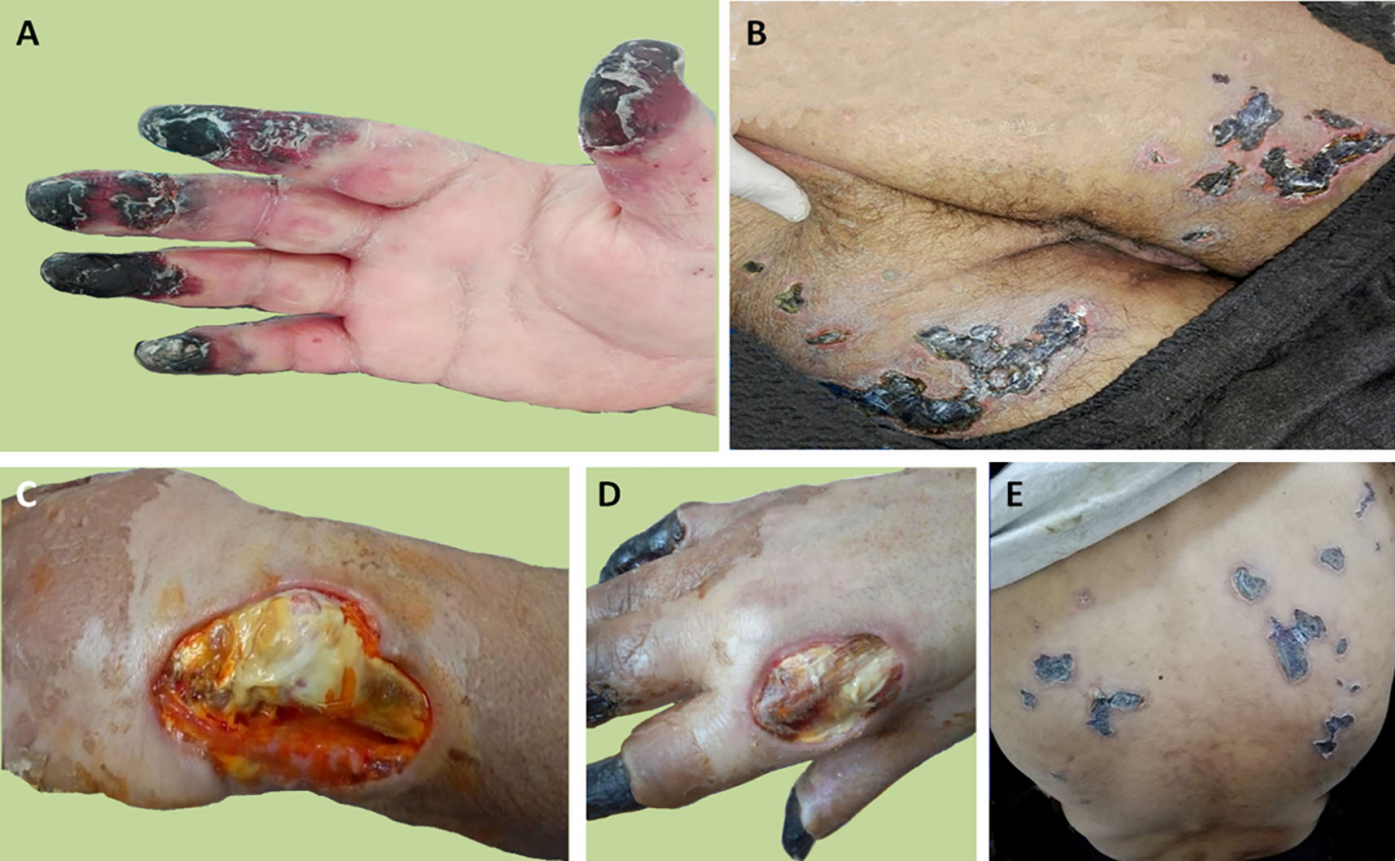
Skin involvement of the patient. A) Extensive digital gangrene. B) Skin necrosis of the thighs. C) Cutaneous substance loss around the left wrist. D) Cutaneous substance loss near the second metacarpophalangeal joint. E) Skin necrosis of the back.

**Figure 2.**
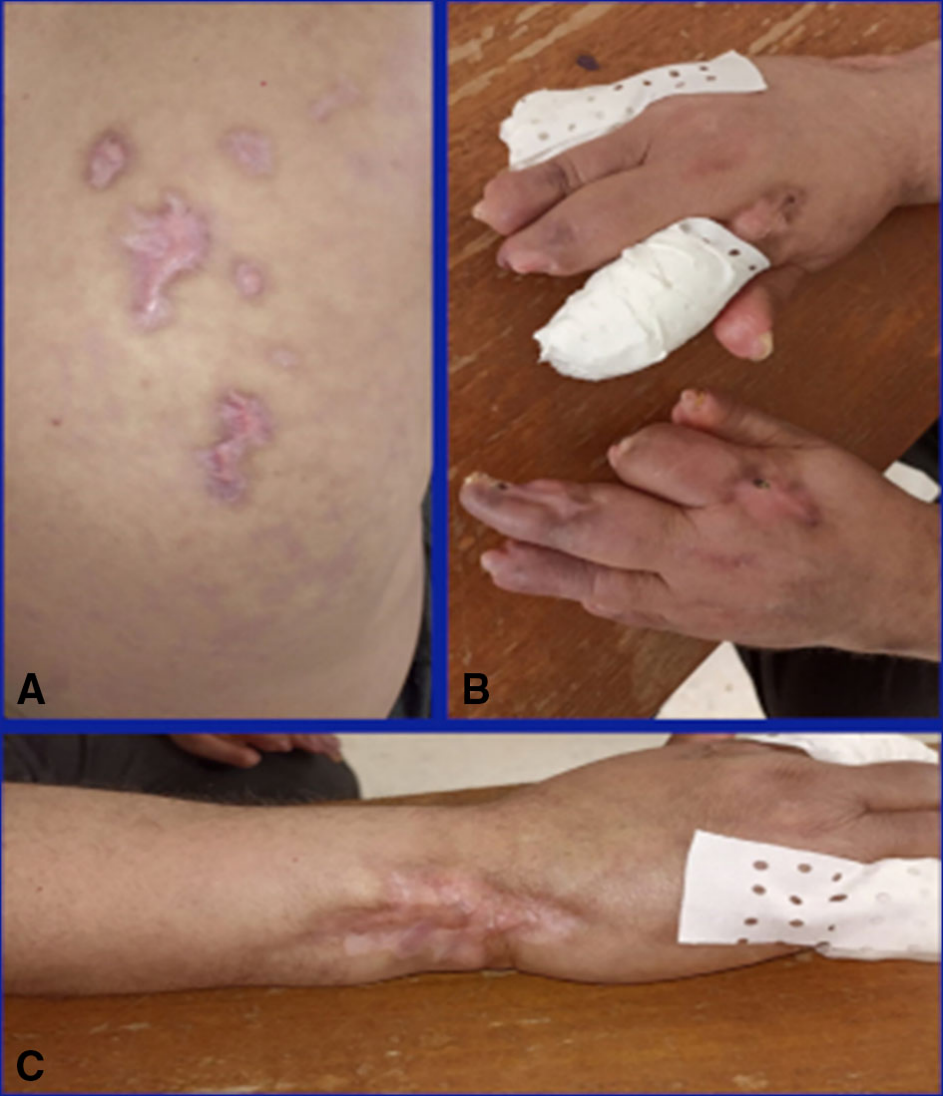
Improvement of the skin involvement after rituximab infusion. A) Improvement of the back skin gangrene. B) Improvement of the digital gangrene. C) Improvement of the left wrist gangrene.

Treatment was initiated with 200 mg/day hydroxychloroquine. For dermal and pulmonary involvement, intravenous (IV) pulse therapy was used with methylprednisolone (PM) (1,000 mg/day for three consecutive days monthly) and cyclophosphamide (CTX) (1 g/month). Calcium blocking agents were also prescribed. A month later, the patient developed digital gangrene and the skin lesions increased ( Figure 1) despite the treatment. Skin lesions were biopsied and the biopsy showed necrotizing vasculitis of SLE in the small vessels. Glucocorticoids (1 mg/kg/day) and anticoagulants (IV heparin infusion 600 UI/kg/day) associated with prostacyclin analog (iloprost, 0.1 mg IV) were therefore prescribed for four days. However, the lesions did not improve. The patient was given two infusions of rituximab (1 g) at a 14-day interval. Secondary infection of the skin lesions occurred and it was successfully treated with antibiotics, leading to a stabilization of digital gangrene, a marked improvement in the majority of vasculitis lesions ( Figure 2), and partial improvement of dyspnea. Glucocorticoids were gradually reduced to 5 mg/day for three months.

### Patient perspective

The patient is currently being monitored on outpatient in the hand surgery department, he reports a marked improvement in skin lesions and dyspnea.

## Discussion

To the best of the authors knowledge, this is the first reported case of a such extensive gangrene as a primary manifestation of late-onset SLE. Digital gangrene is a rare but severe complication of SLE, with a cumulative incidence of 1.3%. It is an inaugural manifestation in rare cases. ^7^ Among 2,684 patients with SLE, digital gangrene occurred in 18 cases, corresponding to a frequency of 0.67% with an average age at the first occurrence of 33.1 years old and an average disease duration of 99.1 ± 60.1 months. ^2^ It was furthermore stated by Rosato *et al.*, that digital ulcers and gangrene are never found as the first presentation in SLE. ^8^ However, some cases of digital gangrene as a primary event in SLE have been reported. ^3^^,^^6^^,^^9^^–^^11^ The majority of cases reported in the literature have proved that digital gangrene occurs in young SLE patients and that the occurrence of this complication in older adult patients is extremely rare. ^2^^,^^6^^,^^9^^,^^10^ To the best of the authors’ knowledge, only three cases of digital gangrene have been reported in patients with SLE at an older age (after 50 years old). The first case was described by Vocks *et al.*, in a 59-year-old female patient. ^12^ The second was reported by Nagai *et al.*, in a 50-year-old female patient followed for SLE since the age of 32 years with cutaneous, hematological, and neurological involvement. ^13^ The third case was reported by Ha-Ou-Nou *et al.*, in a 53-year-old male patient who presented with SLE manifested by extensive digital gangrene. ^11^

The pathophysiological mechanisms of digital gangrene in SLE are complex, involving thromboembolism, premature atherosclerosis, vasospasm, hypercoagulability, and vasculitis. ^2^ Among these manifestations, anti-phospholipid syndrome is a significant risk factor. Jeffery *et al.*, reported that positive antiphospholipid antibody syndrome is one of the causes of critical peripheral ischemia. ^14^ Furthermore, in several reported cases, Doppler ultrasound or even arteriography have revealed digital artery occlusion or stenosis. ^2^^,^^15^^,^^16^ In addition, biopsies have shown moderate and small-vessel vasculitis, even in the presence of normal Doppler ultrasound. ^2^ In our patient, serological markers of antiphospholipid syndrome were negative, upper extremity CT angiography was normal, and skin biopsy showed vasculitis of a small vessel confirming its involvement in the occurrence of this complication. Other risk factors of gangrene have been advanced, including the long duration of the disease (four years), Raynaud’s phenomenon, and high serum CRP. ^2^ The particularity in our patient is that he had no risk factors, and digital gangrene was one of the early signs of the disease. An other particularity of this case is the normality of the complement. this is not uncommon, since the complement may not be consumed in mostly a half of lupus patients. ^17^ Indeed, in an European multinational inception cohort of 200 newly diagnosed SLE patients with active disease, low complement levels were observed at baseline in anly 54%. ^17^ Furthermore, Low complement levels are found even more infrequently in very early and milder disease. ^18^

The severity of this complication (gangrene) justifies the use of early high-dose corticosteroids associated with an immunosuppressive agent. ^2^^,^^9^^,^^15^ Liu *et al.*, reported that the risk of digital amputation declines after early high-dose corticosteroid treatment. ^2^ Ziaee *et al.*, reported a case of a 12-year-old girl treated for digital gangrene with corticosteroids and mycophenolate mofetil with a good outcome. ^15^ Another case of SLE with digital gangrene was treated with corticosteroids and cyclophosphamide, with a significant improvement at the initial infusion. ^11^ For associated anti-phospholipid syndrome, anticoagulation is mandatory. ^11^ Vasodilators and vasoprotective agents should be prescribed in the case of Raynaud’s phenomenon. ^19^ Immunomodulatory treatment, such as rituximab, has also been given to some patients after the failure of corticosteroids and immunosuppressants and it has shown good results. ^14^^,^^20^ This was the case for our patient who did not respond to corticosteroid associated with cyclophosphamide. His lesions improved after two infusions of 1,000 mg rituximab at a 14-day interval. In advanced cases, surgery is necessary. ^19^

## Conclusions

SLE with digital gangrene presenting as an initial symptom is rare, especially in late-onset SLE. Its pathophysiology is complex and multifactorial. Rituximab is a good treatment alternative in case of other treatments’ failure.

## Data availability

### Underlying data

All data underlying the results are available as part of the article and no additional source data are required.

## Consent

Written informed consent for publication of their clinical details and clinical images was obtained from the patient.

## Authors contributions

Dr S B: Conceptualization, Writing- original draft, Supervision, Validation; Dr M B M: Data curation, Writing - original draft; Dr S R: Supervision, Validation; Dr S J: Data curation, Writing - original draft; Dr S R: Supervision, Validation; Dr K Z: Data curation, Writing - original draft; Dr H S: Supervision, Validation; Dr M E: Supervision, Validation.
