# Supplementary material for: Case Report: Extensive digital gangrene as a primary manifestation of late-onset systemic lupus erythematosus
Source: F1000Res. 2022 Sep 7;11:922. Originally published 2022 Aug 10. [Version 2] doi: 10.12688/f1000research.124225.2 (PMC11134139; doi:10.12688/f1000research.124225.2)
Supplement: Peer review history

# Reviewer report: Reviewer response for version 2

**DOI:** 10.5256/f1000research.138015.r270017

**Published:** 2024-05-28

**Authors:** Imad Uthman

**Affiliations:** 1 Division of Rheumatology, Faculty of Medicine, American University of Beirut, Beirut, Lebanon

Excellent, original case report of a rare manifestation of late-onset systemic lupus erythematosus.

 The case description and Discussion sections are very well written and provide a nice review of the pathophysiology of the vasculitis associated with SLE.

 The last section of the discussion also describes thoroughly the different treatment modalities of this complication.

Are enough details provided of any physical examination and diagnostic tests, treatment given and outcomes?

Yes

Is the case presented with sufficient detail to be useful for other practitioners?

Yes

Is sufficient discussion included of the importance of the findings and their relevance to future understanding of disease processes, diagnosis or treatment?

Yes

Is the background of the case’s history and progression described in sufficient detail?

Yes

Reviewer Expertise:

SLE

I confirm that I have read this submission and believe that I have an appropriate level of expertise to confirm that it is of an acceptable scientific standard.

# Reviewer report: Reviewer response for version 2

**DOI:** 10.5256/f1000research.138015.r149772

**Published:** 2022-09-14

**Authors:** Danieli Andrade

**Affiliations:** 1 Department of Rheumatology, University of Sâo Paulo, Sao Paulo, Brazil

The authors addressed all questions properly.

Are enough details provided of any physical examination and diagnostic tests, treatment given and outcomes?

Yes

Is the case presented with sufficient detail to be useful for other practitioners?

Yes

Is sufficient discussion included of the importance of the findings and their relevance to future understanding of disease processes, diagnosis or treatment?

Yes

Is the background of the case’s history and progression described in sufficient detail?

Yes

Reviewer Expertise:

Antiphospholipid Syndrome

I confirm that I have read this submission and believe that I have an appropriate level of expertise to confirm that it is of an acceptable scientific standard.

# Reviewer report: Reviewer response for version 1

**DOI:** 10.5256/f1000research.136407.r147179

**Published:** 2022-08-23

**Authors:** Danieli Andrade

**Affiliations:** 1 Department of Rheumatology, University of Sâo Paulo, Sao Paulo, Brazil

Well written case report of a rare manifestation of SLE: extensive vasculitis as presentation. Very important to have had evaluated antiphospholipid antibodies, that could have been easily missed, and cryoglobulinemia.

 You need to provide the following additional information;

1. Did the patient smoke?
2. Can you please provide the SLE classification criteria that was used for diagnosis?
3. Can you give more details regarding anti-DNA: Was it done by ELISA, and Chritidia? Was it anti-ds DNA? What was the titer? This information is very important to add laboratorial specificity to your case, since anti-Sm was negative. It is very intriguing that complement was normal. You need to make a special consideration regarding it.

Are enough details provided of any physical examination and diagnostic tests, treatment given and outcomes?

Yes

Is the case presented with sufficient detail to be useful for other practitioners?

Yes

Is sufficient discussion included of the importance of the findings and their relevance to future understanding of disease processes, diagnosis or treatment?

Yes

Is the background of the case’s history and progression described in sufficient detail?

Yes

Reviewer Expertise:

Antiphospholipid Syndrome

I confirm that I have read this submission and believe that I have an appropriate level of expertise to confirm that it is of an acceptable scientific standard, however I have significant reservations, as outlined above.

# Response

**Published:** 2022-09-01

**Authors:** Soumaya Boussaid

**Affiliations:** La Rabta Hospital, Tunisia

Dear Reviewer, 

 thank you for your very insightful comments.

1. Did the patient smoke?

No, the patient did not smoke, we have insisted on this point in the following paragraph:" No history suggestive of Raynaud’s phenomenon, diabetes mellitus, smoking, trauma, infection, or chemical exposure was found".

        2. Can you please provide the SLE classification criteria that was used for diagnosis?

 Yes, indeed we specified the SLE classification criteria in the following sentence "Diagnosis of SLE with lung involvement was retained according to the American College of Rheumatology (ACR) classification criteria for SLE. ^6^ "

1. Can you give more details regarding anti-DNA: Was it done by ELISA, and Chritidia? Was it anti-ds DNA? What was the titer? This information is very important to add laboratorial specificity to your case, since anti-Sm was negative. It is very intriguing that complement was normal. You need to make a special consideration regarding it.

You are right, it is important to mention all the data you quoted. in light of your recommendations, we have addeted all information as follow: "Immunological tests were positive for Anti Nuclear Antibodies (ANA) with Title 1:400 (>1/160) speckled and anti-ds DNA dosed by ELISA (with a title of 102,37 UI/ml (NV: 50 UI/ml), anti-SSA 52, and anti-mitochondrial antibody M2. Anti-Smith, anti-SSB, anti-RNP antibodies, and Anti-Neutrophil Cytoplasmic Antibodies (ANCA) were negative."

 For complement normality, we added a paragraph in the discussion section as follows: ". An other particularity of this case is the normality of the complement.  this is not uncommon, since the complement may not be consumed in mostly a half of lupus patients ^17^. Indeed, in an European multinational inception cohort of 200 newly diagnosed SLE patients with active disease, low complement levels were observed at baseline in anly 54% ^17^. Furthermore, Low complement levels are found even more infrequently in very early and milder disease ^18^."
